# A deep semantic network-based image segmentation of soybean rust pathogens

**DOI:** 10.3389/fpls.2024.1340584

**Published:** 2024-03-27

**Authors:** Yalin Wu, Zhuobin Xi, Fen Liu, Weiming Hu, Hongjuan Feng, Qinjian Zhang

**Affiliations:** ^1^ Lushan Botanical Garden, Jiangxi Province and Chinese Academy of Sciences, Jiujiang, China; ^2^ Mechanical Electrical Engineering School, Beijing Information Science & Technology University, Beijing, China; ^3^ Zhongzhen Kejian (ShenZhen) Holdings Co., Ltd, Shenzhen, China

**Keywords:** Asian soybean rust, *Phakopsora pachyrhizi*, deep learning, instance segmentation, mask R-CNN

## Abstract

**Introduction:**

Asian soybean rust is a highly aggressive leaf-based disease triggered by the obligate biotrophic fungus *Phakopsora pachyrhizi* which can cause up to 80% yield loss in soybean. The precise image segmentation of fungus can characterize fungal phenotype transitions during growth and help to discover new medicines and agricultural biocides using large-scale phenotypic screens.

**Methods:**

The improved Mask R-CNN method is proposed to accomplish the segmentation of densely distributed, overlapping and intersecting microimages. First, Res2net is utilized to layer the residual connections in a single residual block to replace the backbone of the original Mask R-CNN, which is then combined with FPG to enhance the feature extraction capability of the network model. Secondly, the loss function is optimized and the CIoU loss function is adopted as the loss function for boundary box regression prediction, which accelerates the convergence speed of the model and meets the accurate classification of high-density spore images.

**Results:**

The experimental results show that the mAP for detection and segmentation, accuracy of the improved algorithm is improved by 6.4%, 12.3% and 2.2% respectively over the original Mask R-CNN algorithm.

**Discussion:**

This method is more suitable for the segmentation of fungi images and provide an effective tool for large-scale phenotypic screens of plant fungal pathogens.

## 1 Introduction

Soybean (*Glycine max*) is one of the most economically efficient crops since it is an important source of food, protein, and vegetable oil. Asian Soybean Rust (ASR) is a globally aggressive foliar disease of soybean plants that can cause up to 80% losses and have a significant impact on production costs in various geographical areas invaded by the pathogen (Lorrain et al., 2019). Fungus *Phakopsora pachyrhizi* is the causal agent of ASR. Infection begins with the deposition of uredospores on soybean leaves, where the rust fungus invades the epidermal cells of the host through the appressorium formed during spore germination and extracts nutrients from the host body (Goellner et al., 2010; Loehrer and Schaffrath, 2011). This fungus can defoliate soybean fields and accelerate maturation with a reduction of seed size and weight and may lead to complete crop failure within a few days (Hartman et al., 2015). Currently, timely fungicide application is the only means of controlling ASR (Saito et al., 2021).

It is important to analyze the characterization of fungi germinating *in vitro* for ASR disease control and research. Researchers have found that automated microscopy-based phenotyping is typically used under genetically or environmentally sensitive conditions to probe the relationship between cell structure and function by unbiased quantification of phenotypic changes in response to perturbations of interest (Liberali et al., 2015; Usaj et al., 2016). Some studies have applied fungal images to the field of drug discovery and development (Calderone et al., 2014; Carolus et al., 2020). By analyzing the morphology and characteristics of fungi, researchers are able to better understand the structure and function of fungi. Statistical information on fungal spores can reveal the degree of resistance and activity of spores to discover new drug candidates and therapeutic options. Large-scale phenotypic screening of multiple compounds acting on fungal spores can identify suitable fungicides and drugs for ASR. However, these drugs usually have to be screened from hundreds of compounds by expert labor, which requires huge processing time. These image-based methods have found their greatest application in the pharmaceutical industry, where they have been used to primary screening stages of drug discovery, drug target validation, early evaluation of toxicity properties and complex multivariate drug profiling (Zanella et al., 2010; Reisen et al., 2015). For example, haploid yeast was treated with drugs that perturb cell wall and the dose-dependent changes in morphology were analyzed to identify drugs that interfere with cell wall synthesis (Okada et al., 2014).

**Figure 1:**
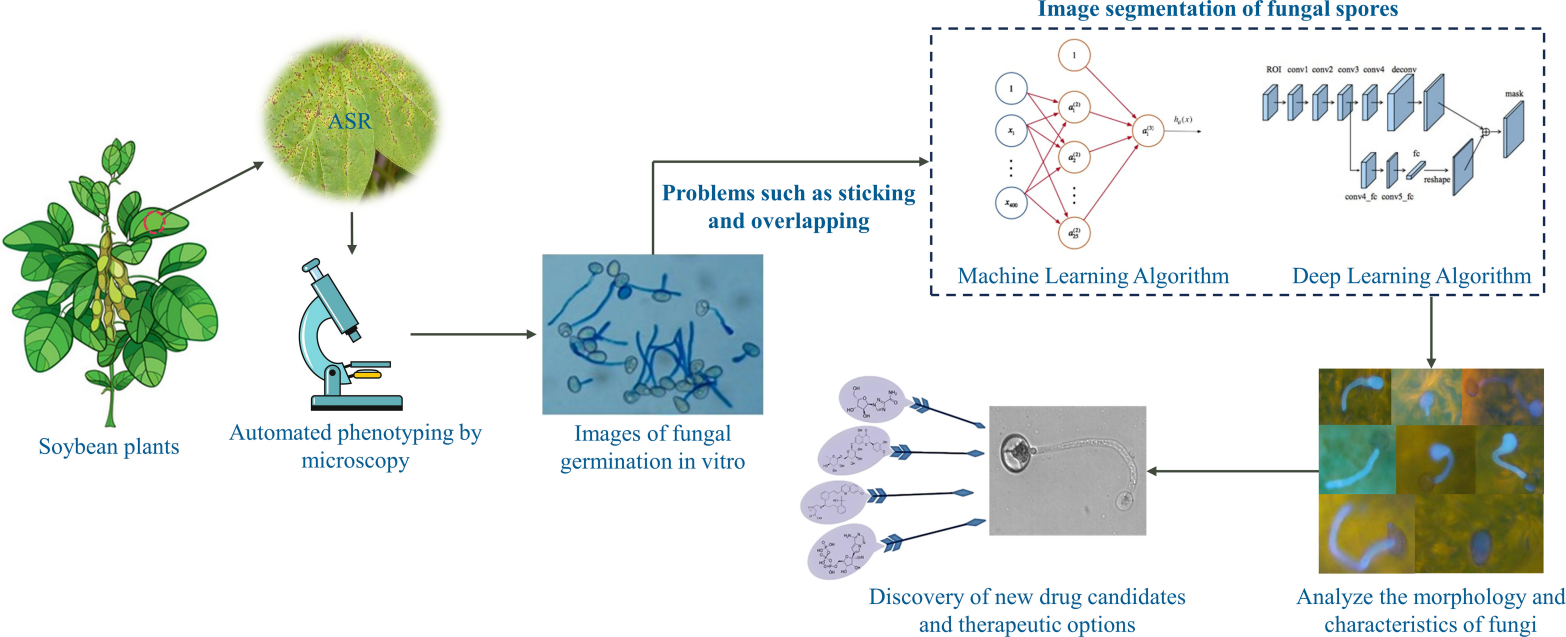
Large-scale phenotypic screening of multiple drugs based on automated fungal segmentation.

As show in **Figure 1**, Segmentation is one of the significant steps of phenotypic screening (Cabre et al., 2021). Accurate image segmentation of fungal spores can characterize phenotypic changes during fungal growth, and accurate segmentation significantly determines the efficiency and effectiveness of drug screening, contributing to the discovery of drugs and agricultural fungicides using large-scale phenotypic screening, as well as to the development of strategies for the control of ASR using biotechnological approaches. Manual segmentation of images is cost-ineffective and time-consuming for expert annotation, and thus is impractical for large data segmentation. More importantly, due to the variability of individuals, manual segmentation can introduce large segmentation errors and biases, so there is a need to find an accurate and efficient automatic segmentation method. Due to the different degree of response of the fungi to different drugs, it appears that the spore morphology of fungi in the process of reproduction appears to have a large morphological variability. Moreover, the interaction between fungi, many fungi overlap, distort and adhere to each other, which can make accurate segmentation difficult. Finally, the collected microscope images have low contrast, and the fungal edges are very blurred and difficult to identify accurately, while some of the images have impurities.

Some microscopy applications use machine learning algorithms, such as those for range thresholding, simple filters, and edge detection based on intensity changes are now widely used (Melo et al., 2019). Traditional image segmentation methods include Otsu’s thresholding (Otsu, 1979), watershed algorithm (Beucher, 1992) and clustering (Coleman and Andrews, 1979). For instance, a Gaussian Separate Degree is used for Otsu method, called as G-Otsu, is proposed to segment anthrax spore images (Zhao et al., 2020). Korsnes et al. (2016) used methods such as mean gradient and morphological processing to detect spore boundaries for spore segmentation, followed by egg shape-fitting techniques to fit spore perimeter. Using K-means method to segment spores of *Puccinia striiformis* f. sp. *tritici* (*Pst*) by clustering pixel values, and isolate touching spores based on the shape and area factors (Lei et al., 2018). However, these classical machine vision methods are sensitive to noise and lack robustness, and usually cannot realize the segmentation of complex shapes.

Deep learning can learn how to extract the features from a large number of samples. Zhao et al. took anthrax spores as the research objects and applied CFL (Constrained Focal Loss) Loss function to DeeplabV3+. Experimentally, this proposed CFLNet* can achieve better performance than original DeepLabv3+ (Zhao et al., 2019). Yang et al. (2020) proposed a Nuclear Segmentation Tool (NuSeT), which assimilates the advantages of semantic segmentation (U-Net) and instance segmentation (Mask R-CNN) and can work with both fluorescent and histopathology image samples. Xie et al. (2021) used Mask Scoring R-CNN network to detect mango disease spores to control and prevent mango disease. Li et al. (2023) proposed an MG-YOLO detection algorithm that introduces Multi-head self-attention in the YOLO backbone and optimizes the network neck and pyramid structure for fast and accurate gray mold spores detection, with a detection accuracy of 0.983 for the improved model and a time spent of 0.009 seconds per image. Zhang et al. (2023) introduced the attention mechanism module (ECA-Net) and adaptive feature fusion mechanism (ASFF) into the feature pyramid structure of YOLO to detect *Fusarium germinate* spores of small targets, and the average recognition accuracy of this model was 98.57%.

Image segmentation includes semantic and instance segmentation. The task of semantic segmentation is to classify each pixel in the image without separating the objects (Long et al., 2015), but this does not apply to our fungal segmentation task because there are a large number of *Phakopsora pachyrhizi* adhering or overlapping in the image, which can cause the touching fungus to not be segmented from each other and cause under-segmentation problems. Instance segmentation is a combination of the object detection and the semantic segmentation, where the object is detected in the image and then each pixel is labeled. Identifying *Phakopsora pachyrhizi* in an image is best viewed as an instance segmentation task (Hariharan et al., 2014). In this paper, we propose an improved Mask R-CNN spore segmentation method to solve these problems and improve the accuracy of spore segmentation. The main objectives include:

Optimization of backbone using Res2net block. by hierarchizing the residual connections in a single residual block, it is possible to achieve a multi-scale characterization of the fine-grained layers and, at the same time, increase the size of the sensory field at each level of the network. The use of Feature Pyramid Grids (FPGs) highlights the importance of deep pyramid representations by improving single-path feature pyramid networks by significantly improving their performance at a similar computational cost. Use CIOU as a bounding box regression loss function to reduce the error.

A new method for fungal spore segmentation is proposed; extensive experiments show that this method achieves better segmentation performance under high density and overlapping conditions.

## 2 Materials and methods

In this section, we first summarize the problems and challenges faced in segmenting target images. Then, the segmentation model is designed and optimized, including the optimization of backbone and the optimization of mask branch.

### 2.1 Data source

In this paper, a spore image dataset was established to characterize the phenotypic transformation of fungi during *in vitro* growth in the presence of different fungicides. We used PerkinElmer’s Opera QEHS high content rotary confocal system to observe fungal spores at different stages of growth and extract high quality images. The rotary disk confocal microscope can scan multi-channel fluorescence signals in a short time, and reduces the influence of detection environment on cells through extremely sensitive confocal imaging and synchronous acquisition.

Fresh leaves with rust organelles that had broken through the leaf epidermis and yellow rust spores were collected from the experimental field and brought back to the laboratory. Firstly, the surface of the diseased leaves was rinsed with running water, and then the leaf surface around the rust organelles was wiped with 75% ethanol, and then the diseased leaves were put into a petri dish with a wet filter paper at the bottom to keep humidity, and then fresh spores scattered around the rust organelles were collected after 1 d. Fresh spores collected were put into a 2 mL EP tube with appropriate amount of sterile water containing 0.3% Tween 80, and shaken well to make a spore suspension. The collected fresh rust spores were put into 2 mL EP tubes, and the spore suspension was made by adding appropriate amount of sterile water containing 0.3% Tween 80 and shaking well. 100µl of the sample was inoculated into the wells of a 96-well plate, and the spores from the 96-well plate were mixed in batches with six solvents: Carbendazim (1 ppm), DMSO (0.1%), PIK-75 (3.3 ppm), Solatenol (0.041 ppm), Solatenol (10 ppm) and TOU-951 (1.1 ppm). After 90 minutes, spores were stained using Calcofluor White solution with KOH and imaging of spores was recorded every 15 minutes one the Opera QEHS at a magnification of 10x to track spore growth status, with a total of 9 time-state data recorded. Hundreds of images of fungal spores at different stages of growth were collected under each time period for each chemical treatment.

**Figure 2:**
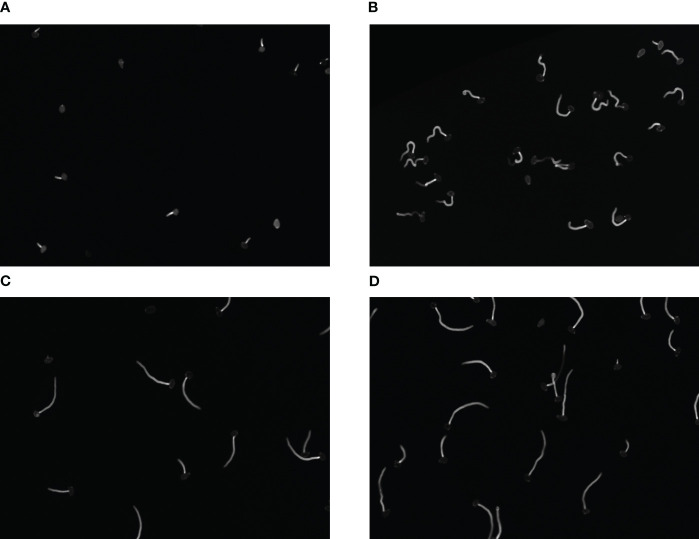
**(A–D)** Respectively represent spores in different growth states.

Each image has a size of 685 × 503 pixels and a pixel range of 8bit, saved in TIF format. We randomly selected 300 images as our dataset which fully contains the various morphology of fungal spores under the action of fungicides, as is show in **Figure 2**. (Due to the different efficacy of drugs acting on fungi, fungal spores vary greatly in phenotype, such as size, length, and number.) The diversity of fungi images collected improves the generalization ability and robustness of algorithm.

**Figure 3:**
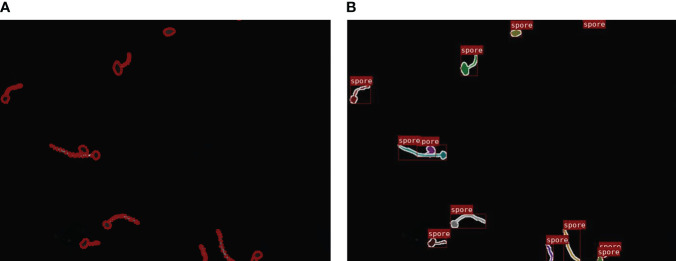
Marking process: **(A)** Data annotation. **(B)** Visualization of the mask image.

Each image in the dataset was manually annotated for network training by the open-source software Labelme (Russell et al., 2008) which labels the pixels of each class. Specifically, to instance segmentation, each single fungus segmentation served as an instance. As is shown in **Figure 3**.

The *Phakopsora pachyrhizi* dataset was divided into training and test sets in a ratio of 80:20, where 240 images were used as the training set (containing 5526 spores) and 60 images were used as the training set (containing 1621 spores). Thereafter, the labeled image instance information is stored in test set and training set json files respectively. The dataset is shown in **Table 1**.

**Table 1:** Number of images and spores in the training and test sets in the dataset under different solution treatments, respectively.

Dataset			Carbendazim	DMSO	Solatenol (0.041 ppm)	Solatenol (10 ppm)	TOU-951
Training set	Images	240	48	48	48	48	48
	Spore instance	5526	1023	1130	970	1112	1291
Test set	Images	60	12	12	12	12	12
	Spore instance	1621	306	312	289	302	412

### 2.2 Instance segmentation methods for spores

Microscopic image segmentation is an intricate task, with the target spores in *Phakopsora pachyrhizi* images encompass fungal spores with variable shapes and the same image mixed with multiple different growth states. An integral fungus consists of 2 parts, the germ tube and the spore. Due to the defects of light and the different sensitivity of different parts of the spore to light, the image of the stained spore has a low contrast, and it is difficult for the naked eye to detect its edges, which improves the difficulty of accurate segmentation. Additionally, growth phenotypes, such as fungal germination count, germ tube length, and growth direction, exhibited significant variations under distinct medicinal treatments. Furthermore, fungi within the same image often appear densely populated, particularly during the later growth stages when exposed to certain solutions. Germ tubes tend to spread across a wide area, resulting in increased instances of crossing, overlapping, and clumping. These challenges have posed difficulties in achieving precise segmentation of *Phakopsora pachyrhizi*.

Image segmentation entails the meticulous classification of pixels into specific categories within an image. In contrast to semantic segmentation, instance segmentation not only segregates diverse objects within an image but also goes a step further by assigning a distinct classification to each individual pixel within the identified instances. This approach facilitates precise localization and differentiation of individual entities within the input image, leading to heightened accuracy in the process.

In the context of microscopic image spore segmentation, the application of instance segmentation techniques offers multifaceted benefits. Beyond effectively addressing challenges arising from spore intersections, overlaps, and adhesions, instance segmentation ensures the isolation of each spore as an autonomous entity, thereby averting any potential information ambiguities. Moreover, this approach excels at precisely determining the spatial coordinates of each spore, encompassing crucial details such as spore boundaries and internal structures. Such precision assumes paramount significance in comprehending the spatial arrangement and density distribution of spores within the image. Through instance segmentation, a nuanced understanding of the spore layout and distribution emerges, thereby enabling more informed analyses and interpretations. Instance segmentation offers the capability to establish distinct units of analysis for each spore, enabling segmentation at an individual level. This approach facilitates the quantification of various attributes like size, shape, color, and additional characteristics inherent to each spore. Consequently, this yields a more comprehensive dataset, enabling a deeper exploration into the intricate nuances of spore variation, interactions, and other pertinent traits. The detailed data acquired through instance segmentation serves as a foundation for conducting exhaustive investigations into the diverse aspects of spore behavior, facilitating enhanced insights and understanding.

Mask R-CNN is a classical top-down two-stage instance segmentation network, which can be considered as the extension of the Faster R-CNN architecture. This network builds upon the original network structure, incorporating additional branches to facilitate the prediction of segmentation masks for each ROI, all the while concurrently performing classification and bounding box regression. The process begins with the input image being fed through the backbone network, resulting in a feature map. This map is then utilized in the Region Proposal Network (RPN) to generate the corresponding anchor boxes. Subsequently, the feature maps linked to each anchor box are homogenized to a consistent size through RoIAlign, ensuring compatibility for further processing. Eventually, this standardized feature map is introduced to a fully connected layer, followed by anchor position refinement executed through regression layers, and class probabilities estimation performed through classification layers. The combination of these processes yields accurate instance segmentation results, with the model generating both precise boundaries and segmentation masks for identified objects.

Researchers are constantly exploring new ways to combine Mask R-CNN with other techniques to improve the performance of segmentation tasks. For example, Seki and Toda (2022) utilized Mask R-CNN to segment lettuce seeds and extract their morphological parameters. Jia et al. (2022) Optimizing Mask R-CNN using the lightweight backbone network MobileNetv3 speeds up the model and meets the storage resource requirements of mobile robots. Chen et al. (2023) incorporated the attention mechanism into the backbone network of Mask R-CNN, which can better detect and segment the tapping area of natural rubber trees under different shooting conditions. Although Mask R-CNN has demonstrated excellent performance in the field of instance segmentation, it still faces great challenges when dealing with data such as spore images, which are characterized by a high degree of overlap and adhesion. In view of this, it is particularly crucial to develop an efficient segmentation strategy for spore image characteristics.

In order to enhancing the precision of spore segmentation, this research introduces an enhanced methodology for spore segmentation using Mask R-CNN. This approach integrates a variant of the Mask R-CNN architecture by incorporating Feature Pyramid Grids (FPG). The single-path feature pyramid network is improved by using FPG, where the feature scale space is represented as a regular lattice of parallel pathways and the pathways are fused together through multidirectional transversal links, which significantly improves the performance of the network with similar computational cost. The backbone network was optimized using an improved backbone, and using Res2Net module by layering residual connections in a single residual block allows for multiscale features at fine-grained layers while increasing the size of the perceptual field at each level of the network.

#### 2.2.1 Feature pyramid grids

**Figure 4:**
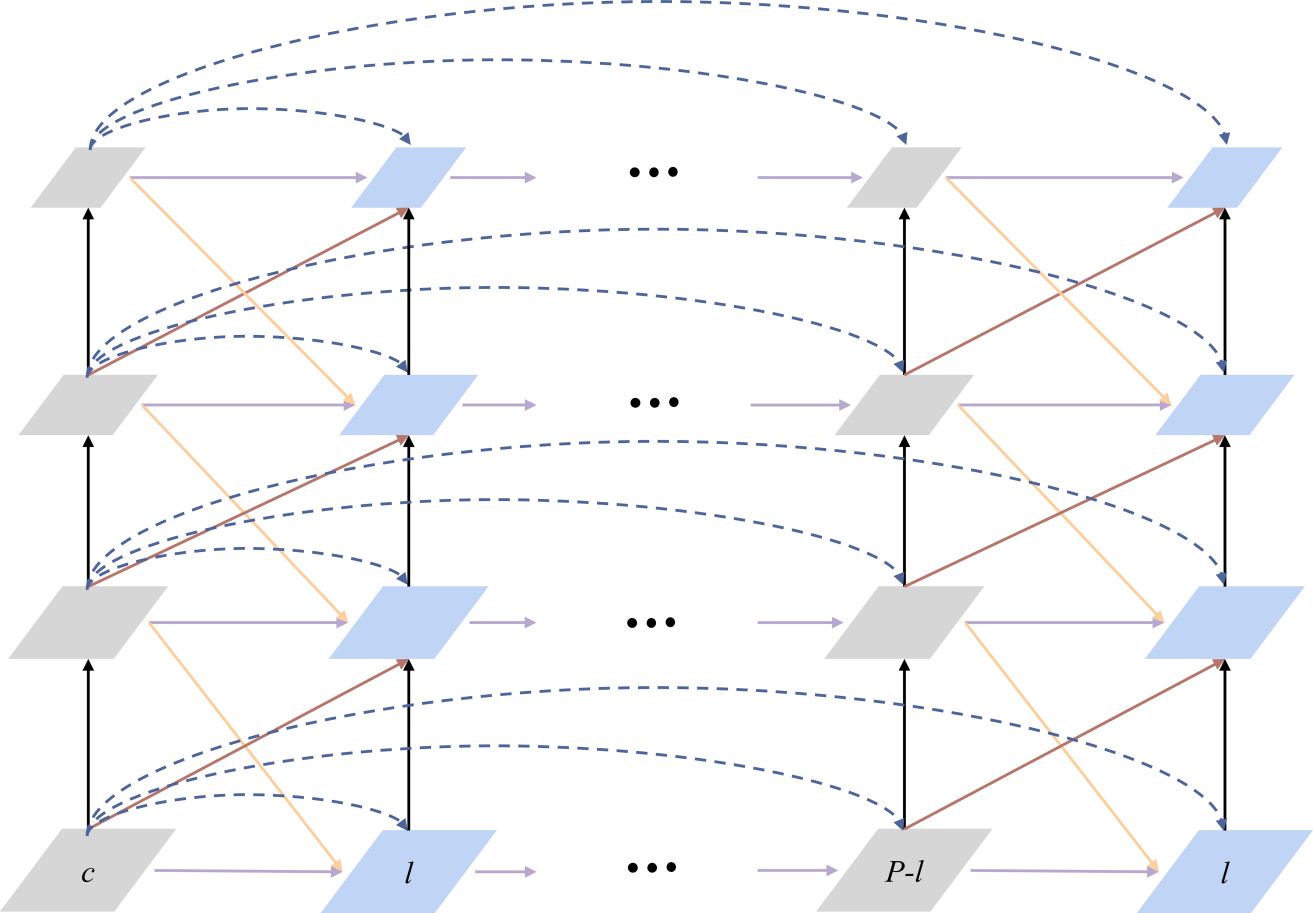
A Feature Pyramid Grid (FPG).

Feature Pyramid Grid (FPG) is an FPN-derived deep multi pathway network as shown in **Figure 4**. The feature scale space of this deep multi pathway feature pyramid network is a fusion of multidirectional lateral connections between parallel paths for information exchange at all levels to build a robust network with high discriminatory power and fine resolution across spatial dimensions. The single pyramid path back-propagates semantic information into the network by successively up-sampling the feature maps. FPG is a parallel extension of the single pyramid, which enriches the multidirectional (semantic) information in the scale space through the lateral connections between feature maps, allowing complex hierarchical features to be learned across scales. Lateral connection has 4 categories. Among them, AcrossSame is the fusion of features of the same level with those in the neighboring paths after using 1*1 convolution. AcossUp uses a convolution of 3*3 stride of 2 to fuse the low-level features of the previous pathway with the high-level features of the next neighboring pathway. AcrossDown fuses the high-level features of the previous pathway to the low-level features of the next neighboring pathway by nearest interpolation convolution with a scaling factor of 2. AcrossSkip uses 1*1 convolutional skip connections between same-level features. Each convolutional block consists of a ReLU, a convolutional layer and a BatchNorm layer, and the fusion function uses element-wise Sum.

#### 2.2.2 Composite backbone

The backbone network in instance segmentation is used to extract features from the input image and determines the feature representation capability of the model. For densely distributed fungi in the image, the large appearance of spores especially at the late stage of chemical treatments processing elevates the segmentation difficulty. In order to obtain better segmentation performance under high density and overlapping conditions, we optimize the backbone network based on FPN using Res2Net (Gao et al., 2019) fusion FPG as a composite backbone. Compared with ResNet, Res2Net adds small residual blocks to extract features with different receptive fields and multiple scales, so that the network can learn multiple features with different scales, in order to promote the communication of multi-scale features.

**Figure 5:**
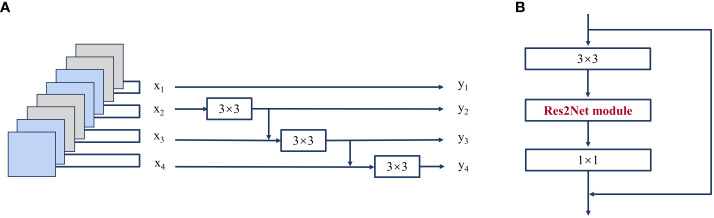
**(A)** Res2Net module (scale=4); **(B)** Res2Net block.


The Res2Net structure is shown in **Figure 5**. In the Res2net module, the input features are categorized into s subset, denoted as xi, i∈{1,2,…,s}; the number of channels of the feature map in each group is 1/s of the number of channels of the input feature map. Then, each set of feature maps undergoes a 3×3 convolution (denoted as K_i_), except for x1. Starting from x3, the feature map xi of the ith group is first summed with the K_i-1_ output of the previous group, and the result of the sum is subjected to the K_i_ operation. The whole process is represented in Formula (1).

(1) $$y_{i}=\{\begin{array}{cc} x_{i} & i=1\\ K_{i}\left(x_{i}\right) & i=2\\ K_{i}\left(x_{i}+y_{i-1}\right) & 2<i\leq s \end{array}$$

where y_i_ is the output of the module that is fed into the next convolutional layer. s is *scal*, which serves as the number of parameters controlling the dimensionality of the dimensions, and the larger s is, the better the multidimensional characterization. In this study, the s value size of 26 is used as the Res2Net block, which is used to modify the ResNet structure in the FPN.

Meanwhile, drawing on the idea of FPG, the improved backbone is combined with FPG to represent the feature scale space as a regular lattice of parallel pathways, and the pathways are fused together by multidirectional transverse links, improving the single-path feature pyramid network, which significantly improves the performance of the network with similar computational cost.

**Figure 6:**
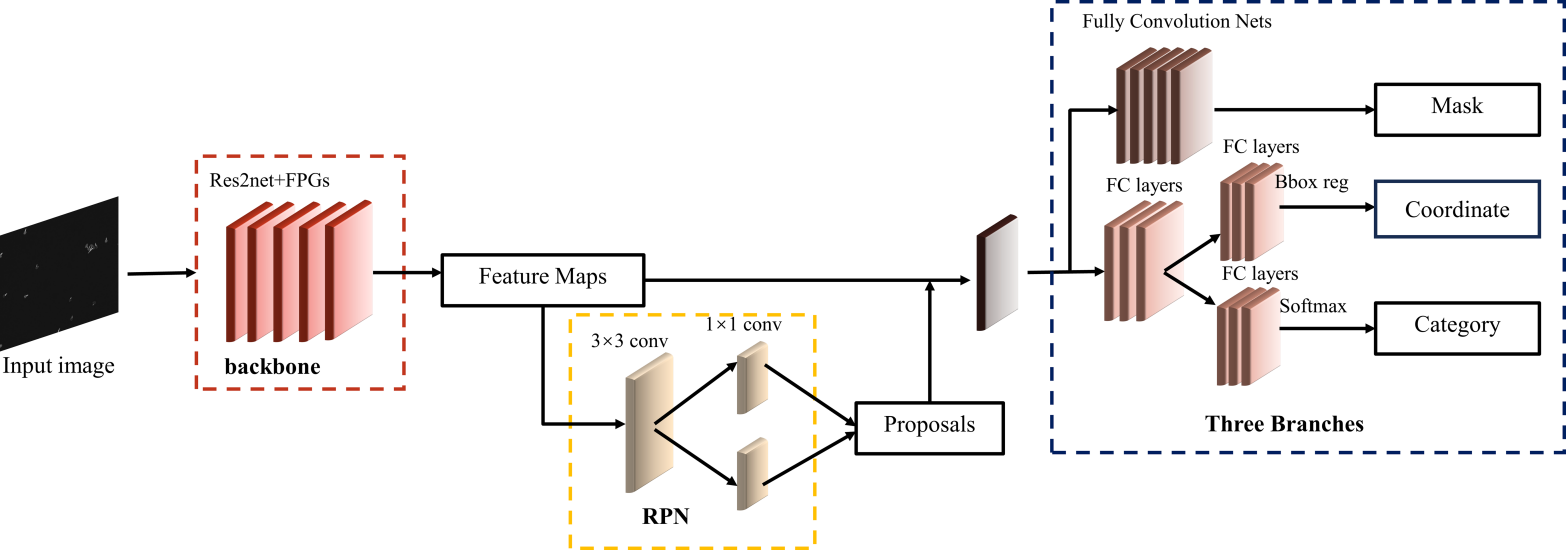
The overall framework of the improved Mask R-CNN.

The design of Backbone is shown in **Figure 6**. The bottom-up C2, C3, C4 and C5 are Res2Net module layers, and the stride of each layer is 2. The number of channels of the structural layers output from C2 to C4 are adjusted using 1*1 convolution, which produces P21, P31, P41 and P51, respectively, with P61 being derived from P51. To enhance computational efficiency, some simplifications were made in the lateral connections of FPG. Specifically, the AcrossSkip connection was removed, and a subset of the AcrossSkip, SameUp, and AcrossDown connections were omitted. We retained half of the triangular structure in the lateral connections, and in our experiments, we opted for P=9 paths to enrich the network’s capabilities.

#### 2.2.3 Loss function

In the whole improved network structure, the corresponding loss function consists of five parts, which are: the RPN’s classification result prediction *L_R_*_-_ *_cls_*, its bounding box regression prediction *L_R_*_-_ *_box_*, alongside the final classification result prediction *L_cls_*, final bounding box regression prediction *L_box_*, and the final mask image prediction *L_mask_*. Loss function is calculated by Formula (2).


(2) $$L=L_{R-cls}+L_{R-box}+L_{cls}+L_{box}+L_{mask}$$

Classification loss *L_cls_* computes the loss of class probability using Cross Entropy.

*L_mask_* uses the Binary crossentropy loss function, calculated by the Formula (3).


(3) $$L_{mask}=-\sum_{y}y\log(1-\hat{y})+(1-y)\log(1-\hat{y})$$

where *y* denotes the binarized ground truth, $\hat{y}$ denotes the predicted segmentation result after binarization.

Edge information is very important for instance segmentation, and they can characterize the instance well. Mask R-CNN begins by utilizing the smooth*_L_*_1_ function for calculating edge loss in target detection. Within this approach, losses for the four coordinate points are computed separately and aggregated to derive the ultimate edge loss. Despite assuming independence among the four points, there exists a certain degree of correlation among them in reality. The process of assessing box detection involves employing Intersection over Union (IoU), which differs from the regression coordinate box derived from the four points. Multiple detection boxes might yield identical smooth*_L_*_1_ Loss values despite differing IoU values. To address this disparity, IoU Loss was introduced as a solution.

However, researchers and scholars soon found that IoU Loss has a drawback: when the prediction box does not intersect the target box, the loss function is not derivable. This problem makes the boundary information ignored in the prediction, and inaccurate edge detection occurs in the experiment, which affects the accuracy of segmentation. In order to meet the accurate segmentation of high-density spore images and improve the sensitivity of boundary segmentation, this paper optimizes the loss function and adopts the *L_CIoU_* loss function as the loss function of the bounding box regression prediction, which accelerates the convergence speed of the model and makes the results of boundary segmentation more accurate.

The *L_CIoU_* calculates the discrepancy between the predicted bounding box and the ground truth. Its definition is outlined in Formula (4).


(4) $$\{\begin{array}{l} L_{CIoU}=1-IoU+\frac{\rho^{2}\left(b,b^{gt}\right)}{e^{2}}+\frac{v^{2}}{\left(1-IoU\right)+v}\\ IoU=\frac{\left|b\cap b^{gt}\right|}{\left|b\cup b^{st}\right|} \end{array}$$

Here, v signifies the alignment of the two frame aspect ratios, while b and $b^{gt}$ denote the center coordinates of the prediction and actual boxes respectively. ρ represents the Euclidean distance between their center points, indicative of the diagonal span of the smallest enclosed area containing both boxes. IoU stands for the Intersection over Union, representing the ratio between the shared area and the combined area of the predicted and actual bounding boxes.

In summary, the loss function used in this paper takes into account the error factor between the predicted value and the true value, which improves the convergence rate of the model, and the optimized network is more accurate in terms of error, and more flexible and feasible.

## 3 Results and discussion

### 3.1 Evaluation metrics

In this section, we present the key metrics used to measure the performance of spore instance segmentation. By leveraging these metrics, we can objectively analyze and showcase the strengths of our method, while also enabling a comprehensive comparison with existing instance segmentation techniques.

Average precision (AP) and average recall (AR) are the main evaluation metrics currently used in the field of object detection and instance segmentation. These metrics depend on two different segmentation masks: a ground truth segmentation mask labelled by experts and an output segmentation mask predicted by the network. Calculating AP and AR requires first calculating Precision, Recall, and IoU (Intersection over Union), as shown in Formulas (5–7).


(5) $$Precision=\frac{TP}{TP+FP}\times 100\%$$

(6) $$Recall=\frac{TP}{TP+FN}\times 100$$

TP is true positive which means the number of correctly detect fungal areas, FP as false positive means the number of incorrectly detect fungi areas and FN is false negative which means the number of fugal areas incorrectly detected as background. Precision represents the proportion of TP in the predicted fugal areas and Recall means the proportion of TP in the true fungal areas.


(7) $$IOU=\frac{\text{ target }\cap \text{ prediction }}{\text{ target }\cup \text{ prediction }}\times 100\%$$

IoU is the metrics to evaluate segmentation accuracy in one category, which calculate the intersection over union between predicted object and ground truth object.

Since the spore image is a small object, we select four metrics, AP, AP50, AP75, and AR, for network performance evaluation. Where AP is io ranging from 0.5 to 0.95 with a step rate of 0.05, AP50 is an iou threshold of 0.50, and AP75 is an iou threshold of 0.75. The higher these values are, the more desirable the instance segmentation model is (Tong et al., 2020).

AR calculates the average recall at different thresholds, how many real objects are correctly detected by the model. AR is the maximum recall of a given fixed number of detections per image, averaged over all IoU and all categories. Since there is only one type of spore, the category is 1. In this study, AR was calculated and averaged over 10 IoU thresholds between 0.5 and 0.95, as shown in Formula (8).


(8) $$p_{\text{interp}}(r)=\underset{\tilde{r}:\tilde{r}\geq r}{\max}p(\tilde{r})$$

where $p\left(\tilde{r}\right)$ is the measured precision at recall $\tilde{r}$ .

AP is calculated by the Formula (9).


(9) $$AP=\frac{1}{11}\sum_{r\in \{0,0.1,\ldots 1\}}p_{\text{interp}}(r)$$

### 3.2 Implementation details

The computational environment for this study utilizes Python 3.7.3 and Ubuntu 18.04 LTS, employing Jupyter Notebook as the editor. The integrated model outlined above was constructed on an Intel(R) Core (TM) i7-12700H (20 CPU) with a 2.30GHz processor, 16 GB of DDR4 RAM, and three graphics cards: two discrete graphics cards (NVIDIA GeForce RTX 3060 laptop GPU with 6023 MB, NVIDIA GeForce RTX 3080 Ti with 12108 MB) along with one integrated graphics card (Intel(R) Iris(R) Xe graphics card with 128 MB), which were utilized for training and testing.

The instance segmentation model was trained with stochastic gradient descent (SGD) method, batch size was set to 4, momentum factor was 0. 9, the initial learning rate was 0.08, and for each epoch, the learning rate changed to 0.9 times of the previous one. The total number of epochs for model training is 100, and when training for the first 60 epochs, the pre-feature extraction network is frozen, and only the neck network and the detection head network are trained in order to improve the training speed of the network model.

### 3.3 Performance comparison with state-of-the art methods and visualization analysis

**Figure 7:**
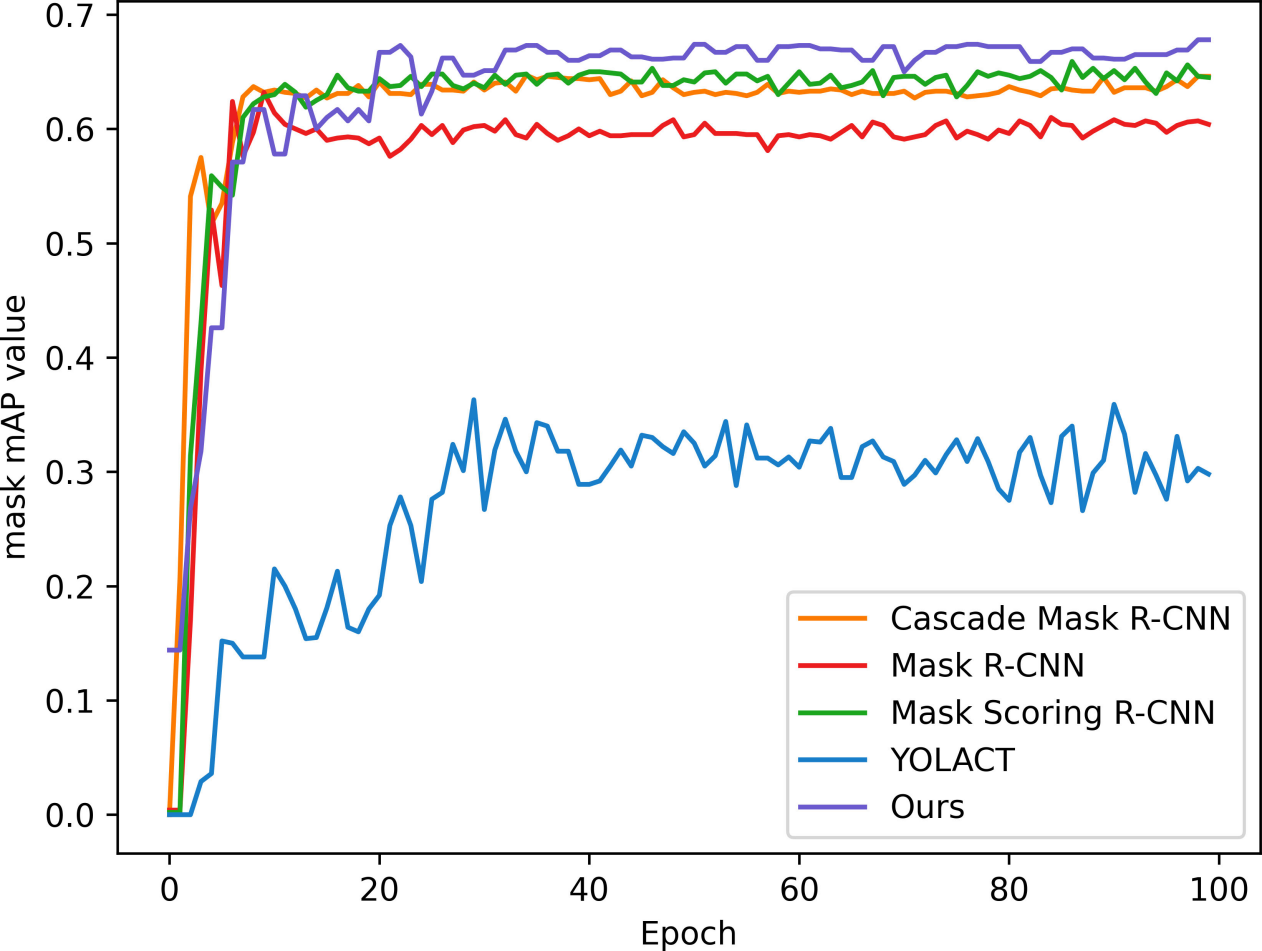
Mask segmentation mAP of the model.

**Figure 8:**
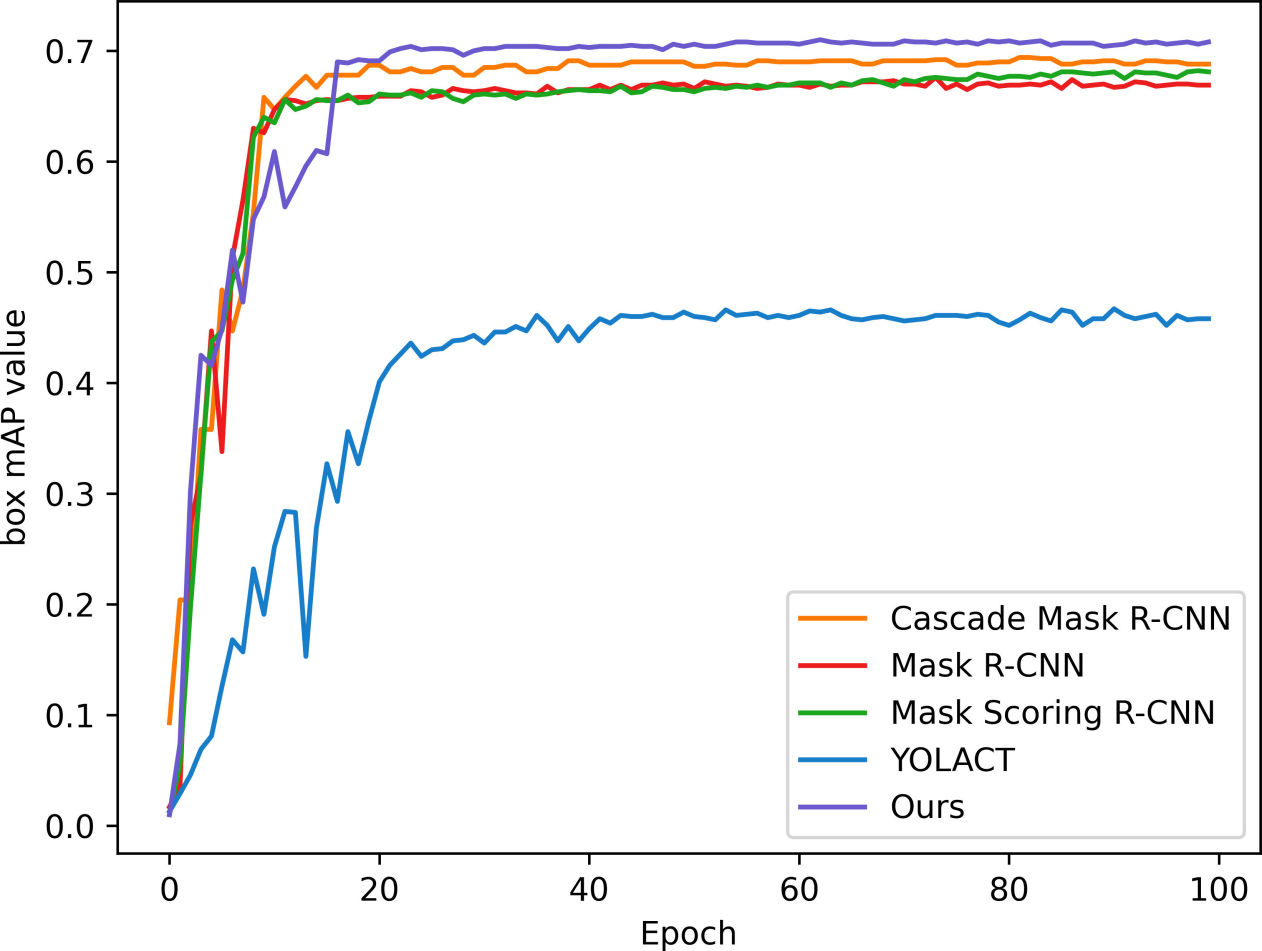
Box detection mAP of the model.

In order to verify the effectiveness and accuracy of the model in spore instance segmentation, we use the same spore dataset under the same training environment and experimental configuration, and analyze them in comparison with Mask R-CNN, Mask Scoring R-CNN (Huang et al., 2019), YOLACT (Bolya et al., 2019) and Cascade Mask R-CNN (Cai and Vasconcelos, 2019) models. The parameters introduced in 3.2 are used as evaluation indexes to compare the performance with several other methods, and the experimental results are shown in **Table 2**. All algorithms are trained for 100 epochs, and after each epoch is completed, the mAP values for mask segmentation and box detection are calculated, as shown in **Figures 7** and **8**.

**Table 2:** Evaluation results of serious models.

Models	box_mAP	box_mAP_50	box_mAP_75	mask_mAP	mask_mAP_50	mask_mAP_75
Cascade Mask R-CNN(ResNet-101)	0.688	0.911	0.802	0.646	0.796	0.696
Mask R-CNN(ResNet-101)	0.669	0.938	0.754	0.604	0.757	0.619
Mask Scoring R-CNN(ResNet-101)	0.681	0.929	0.787	0.645	0.785	0.695
YOLACT(ResNet-101)	0.458	0.836	0.49	0.298	0.442	0.036
Ours	0.712	0.936	0.809	0.678	0.828	0.724

In this application, detection accuracy refers to the detection of spore individuals from complex environments, detection accuracy refers to the model correctly identifying and localizing spore instances in complex environments, and segmentation accuracy is concerned with the model correctly segmenting each spore at the pixel level, and segmentation accuracy is as important as detection accuracy. As can be seen from the table, our improved algorithm outperforms these classical instance segmentation algorithms in both segmentation accuracy and detection accuracy. From the parameter comparison, the detection accuracy of the model is 0.712 and the segmentation accuracy is 0.618, which are 3.5% and 5% better than the existing optimal methods, respectively. As can be seen in **Figures 7** and **8**, when the training epoch is less than 20, the advantage of the method is not obvious. However, as the training epoch increases, the detection mAP of our proposed method clearly outperforms these state-of-the-art methods. Our model is more powerful because we not only optimize the backbone network to efficiently extract global and local features; we also introduce a deep multipath feature pyramid network to construct fine-resolution features with strong semantic information; all of these improvements greatly improve the robustness of the CNN to geometric transformations of the target. Our model is able to explore the complex nuances of spore variants, interactions and other related features in greater depth, understand the spatial arrangement and density distribution of spores in an image, effectively resolve spore crossings, overlaps and adhesions, and ensure that each spore is separated as an independent entity.

**Figure 9:**
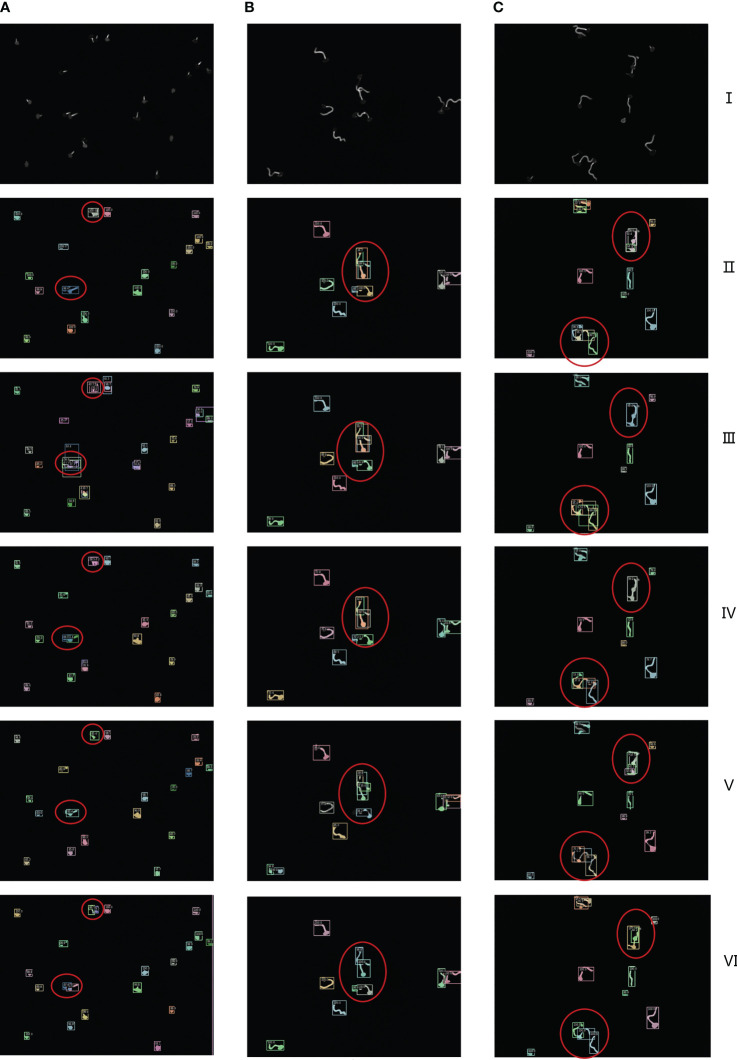
I is the original image of spores, II to VI are Cascade Mask R-CNN, Mask R-CNN, Mask Scoring R-CNN, YOLACT, ours network model visualization and analysis images respectively. **(A–C)** are the segmentation results of three randomly selected spore images under different networks.

In order to highlight the superiority of the proposed architecture more intuitively, a visual comparative analysis between the current networks and ours is carried out. As shown in **Figures 9A–C**, in the figure, are three different growth patterns of spores, where I is the original spore images, and II to VI are Cascade Mask R-CNN, Mask R-CNN, Mask Scoring R-CNN, YOLACT, and ours, respectively.

**Figure 10:**
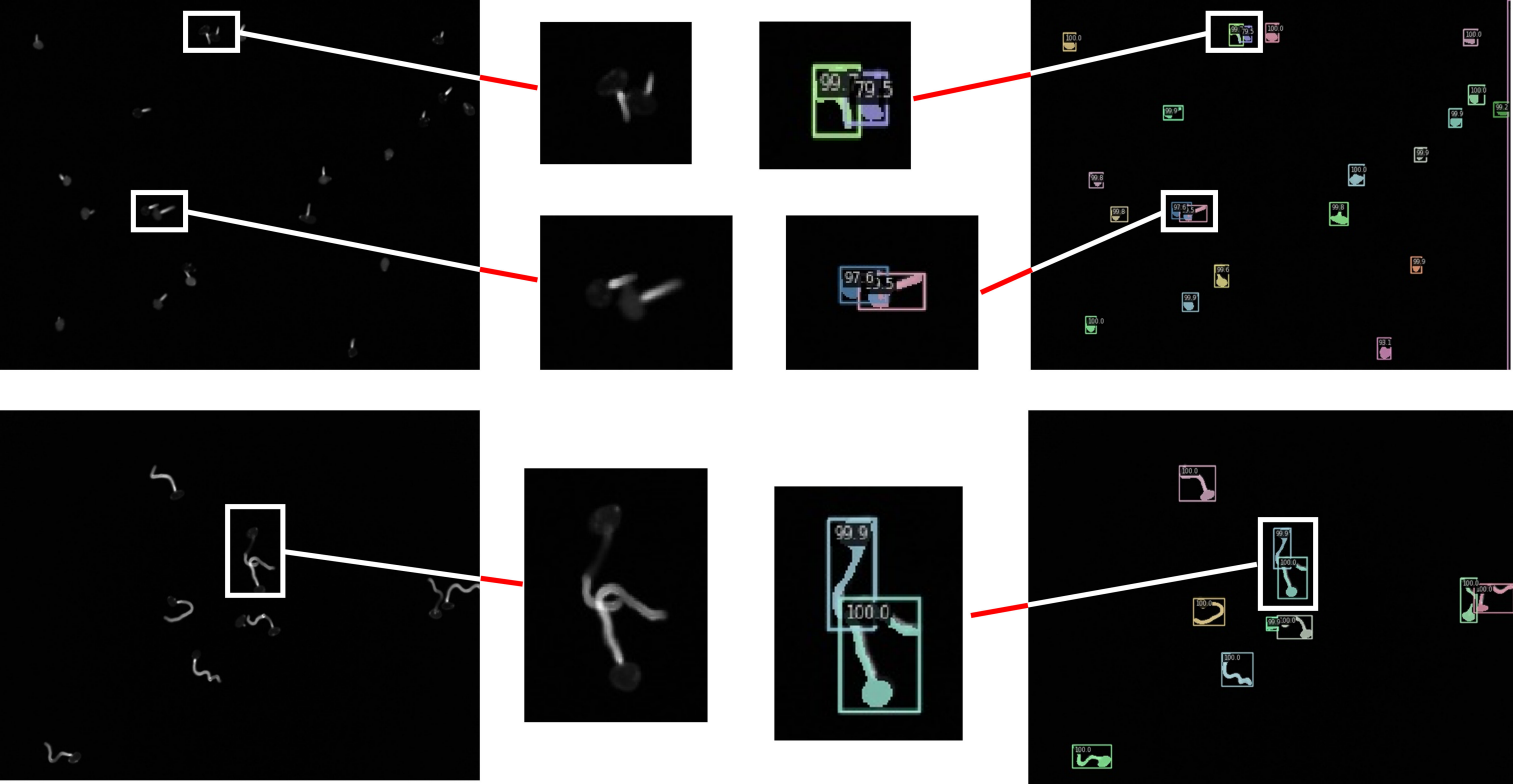
Visualization results of the target network.

As can be seen from the confidence level of the anchor box and the box in the figure, the example segmentation results of these advanced networks for a single scattered distribution of spore images are more general. As can be seen from the red circles in the figure, under-segmentation and over-segmentation occur for spore cross, adhesion and overlapping part segmentation with low confidence and lack of refinement and edge processing. In sharp contrast, our network model generated finer detection segmentation images. To further demonstrate the visual analysis results of this network, we performed a zoom-in comparison, as shown in **Figure 10**.

We chose two images with sparse and tight spore distribution, and both images contain cross overlapping spore features. As can be seen from the figure, the model uses CIoU loss optimization to obtain the optimal prediction box, so that the box detection part can quickly and accurately find the differences between spore individuals with high confidence. At the same time, the model effectively solves the problem of poor robustness of fuzzy pixel segmentation, and the mask segmentation part not only refines the overall segmentation, but also greatly improves the edge segmentation accuracy, so that the cross-overlapping spore images are separated as separate individuals.

### 3.4 Ablation study of improved models

Based on the improved Mask R-CNN model proposed in this paper, ablation experiments were conducted to compare the experimental results of the original model with the improved ResNet-101, ResNet-101+FPG, Res2Net-101+FPG, and Res2Net+FPGs, as shown in **Table 3**. **Table 3** shows the experimental results of different backbone networks: after adding FPG to the original network, the detection accuracy of the model decreases significantly and the instance segmentation accuracy decreases, but the segmentation accuracy is greatly improved; after replacing ResNet-101, the detection accuracy, segmentation accuracy and accuracy are significantly improved and exceed the original model, which indicates that each module of the improvement has a positive effect on instance segmentation. Finally, the best performing model was found to come from the combined effect of the two improved modules, which improved the detection accuracy, segmentation accuracy, and instance segmentation accuracy by 6.4%, 12.3%, and 2.2%, respectively, compared to the Mask R-CNN model, proving that these improvement strategies of the model are effective.

**Table 3:** Evaluation results of ablation experiments.

Models	box_mAP	mask_mAP	acc	#param.	GFLOPs
Original Mask R-CNN(ResNet-101)	0.669	0.604	95.99	63.388M	302
Mask R-CNN(ResNet-101+FPG)	0.621	0.67	94.72	63.274M	147
Mask R-CNN(Res2Net-101+FPG)	0.69	0.678	96.77	63.951M	152
Ours	0.712	0.678	98.144	63.951M	154

## 4 Conclusion

In this study, an improved Mask R-CNN method is proposed to accomplish the task of segmentation of densely distributed and overlapping crossed *Phakopsora pachyrhizi* micro-images. The method is optimized and improved from the original MaskR-CNN. Firstly, the res2net was used to replace the backbone network of the original Mask R-CNN by layering the residual connections in a single residual block and then combining it with FPG in order to improve the fine resolution and high-resolution capability, strengthen the feature extraction capability of the network model, and enhance the detection accuracy. Secondly, for the problem of inaccurate edge detection of the original model, the loss function is optimized, and the CIoU loss function is adopted as the loss function of the boundary box regression prediction, which accelerates the convergence speed of the model, meets the accurate segmentation of high-density spore images, and improves the sensitivity of boundary segmentation. Compared with the original model, it is more robust and further improves the accuracy of instance segmentation. In summary, the proposed model can better detect and segment spores under various conditions.

However, this study suffers from an insufficient number of samples in the dataset, and the accuracy of detection and segmentation in the case of spore stacking with a large number of anomalies needs to be further improved. In the follow-up work, collecting and labeling more spore clusters with complex shapes should be considered to expand the spore dataset under different overlap types. Meanwhile, in order to improve the performance of the network, the effect of spore morphology such as length, width and area on segmentation can be deeply investigated, and its features can be fused with image information and input into the segmentation network. Finally, in future research, the scheme proposed in this paper needs to be installed and applied in real scenarios to validate the performance of the model and algorithm. The technique can be applied to perform automatic segmentation of images on microscopes to facilitate the discovery of new drug candidates and the discovery of therapeutic options. Simultaneously, it offers valuable insights to the fields of agriculture, ecology, and medicine, enhancing our understanding and management of fungal-related issues, including disease transmission and ecological balance.

## Data availability statement

The raw data supporting the conclusions of this article will be made available by the authors, without undue reservation.

## Author contributions

YW: Validation, Writing – review & editing. ZX: Writing – original draft. FL: Data curation, Writing – review & editing. WH: Investigation, Writing – review & editing. HF: Writing – review & editing. QZ: Supervision, Writing – review & editing.
